# Proteomic Analysis of MeJa-Induced Defense Responses in Rice against Wounding

**DOI:** 10.3390/ijms20102525

**Published:** 2019-05-22

**Authors:** Laura Bertini, Luana Palazzi, Silvia Proietti, Susanna Pollastri, Giorgio Arrigoni, Patrizia Polverino de Laureto, Carla Caruso

**Affiliations:** 1 Department of Ecological and Biological Sciences, University of Tuscia, 01100 Viterbo, Italy; lbertini@unitus.it (L.B.); s.proietti@unitus.it (S.P.); 2 Department of Pharmaceutical and Pharmacological Sciences, University of Padova, 35131 Padova, Italy; luana.palazzi@unipd.it; 3 Institute for Sustainable Plant Protection, National Research Council of Italy, Sesto Fiorentino, 50019 Florence, Italy; susanna.pollastri@ipsp.cnr.it; 4 Department of Biomedical Sciences, University of Padova, 35131 Padova, Italy; giorgio.arrigoni@unipd.it; 5 Proteomics Center of Padova University and Azienda Ospedaliera di Padova, 35131 Padova, Italy

**Keywords:** MeJA, priming, rice, proteomics, ROS, chlorophyll fluorescence imaging

## Abstract

The role of jasmonates in defense priming has been widely recognized. Priming is a physiological process by which a plant exposed to low doses of biotic or abiotic elicitors activates faster and/or stronger defense responses when subsequently challenged by a stress. In this work, we investigated the impact of MeJA-induced defense responses to mechanical wounding in rice (*Oryza sativa*). The proteome reprogramming of plants treated with MeJA, wounding or MeJA+wounding has been in-depth analyzed by using a combination of high throughput profiling techniques and bioinformatics tools. Gene Ontology analysis identified protein classes as defense/immunity proteins, hydrolases and oxidoreductases differentially enriched by the three treatments, although with different amplitude. Remarkably, proteins involved in photosynthesis or oxidative stress were significantly affected upon wounding in MeJA-primed plants. Although these identified proteins had been previously shown to play a role in defense responses, our study revealed that they are specifically associated with MeJA-priming. Additionally, we also showed that at the phenotypic level MeJA protects plants from oxidative stress and photosynthetic damage induced by wounding. Taken together, our results add novel insight into the molecular actors and physiological mechanisms orchestrated by MeJA in enhancing rice plants defenses after wounding.

## 1. Introduction

Plants are exposed to a variety of external factors that unfavorably affect their growth and development, and are generally classified into biotic (microbial pathogens and insect herbivores) and abiotic (extreme temperature, water logging, drought, high salinity or toxic compounds, etc.) stresses. Adaptation to these environmental stresses is essential for survival, growth and reproduction [1]. Among the defense strategies that plants have evolved, some are constitutive whereas other are induced in response to stimuli, thus being more specific [2]. It is widely recognized that the identification of elicitors triggers the activation of peculiar subsets of defense responses [3]. Furthermore, plants are able to recognize non-self molecules or signals from their own damaged cells and consequently to activate an efficient immune response against the stress they encounter [4,5,6]. It has been shown that phytohormones such as salicylic acid (SA), jasmonic acid (JA), ethylene (ET), abscisic acid (ABA), cytokinin, brassinosteroids and auxin are the main players in coordinating signaling networks involved in the adaptive response of plants to its (a)biotic environment [6,7]. These signal-transduction pathways in turn activate large suites of genes, including those coding for transcription factors, enzymes involved in the production of plant toxins, plant volatiles and reactive oxygen species (ROS) [8]. Generally, SA induces defense responses against biotrophic pathogens, whereas JA and ET are important hormonal regulators of induced reaction against necrotrophic pathogens [6]. Moreover, it has been shown that either JA or ABA induce plant defenses against herbivorous insects, and that both JA and its methyl ester (MeJA) are key components of a wound signal transduction cascade in plants [6,9]. Indeed, application of exogenous JA induces the expression of genes, such as phenylalanine ammonia lyase and proteinase inhibitors, known to be responsive to wounding [10]. Furthermore, using defective tomato mutants in both JA biosynthesis and perception in grafting experiments, it has been further demonstrated that JA or one of its derivatives may also act as a long-distance transmissible wound signal [10,11,12,13].

Recent evidences show that plants can be primed for more rapid and robust activation of defense response to biotic or abiotic stresses [14,15]. Defense priming is considered to be an adaptive, low-cost defensive strategy since defense responses are not, or only slightly and transiently, activated by a given priming agent. Conversely, defense responses are activated in a faster, stronger, and/or more persistent manner following the perception of a later challenging signal [15]. Effectively, primed plants possess molecular mechanisms that allow them to memorize previous priming events and generate memory imprints during the establishment of priming [16,17,18,19].

The primed state can be induced by a pre-exposition of the plants to low doses of natural or synthetic (a) biotic stress inducers, among which are chemical compounds (hormones, pipecolic acid, hexanoic acid, volatile organic compounds), pathogens, insect herbivores, beneficial microorganisms or environmental cues [3,20,21,22,23]. To date, induction of priming by chemicals has been observed in many plant species, such as parsley, tobacco, *Arabidopsis thaliana* as well as in many others monocots and dicot species [24,25]. The mediation of hormones in the primed responses is mainly restricted to SA, ET, JA and ABA [16,26,27]. Among them, JA was studied in relation to resistance induction, demonstrating that Arabidopsis plants primed with JA showed protection and reduction of infection symptoms by *P. cucumerina* and *A. brassicicola* [28]. The role of JA as a priming hormone was also studied in rice following *Rhizoctonia solani* infection [29]. Moreover, Methyl Jasmonate (MeJA)-induced priming was studied in the herbaceous monocotyledon *Calla lily*, infected with the necrotrophic bacterium *Pectobacterium carotovorum*, highlighting decreased necrosis in infected plant tissues [30]. Unraveling the molecular basis of priming has recently received increasing attention [17,21,31,32]. Depending on the nature of the priming agent and the stressor, priming can involve diverse mechanisms. Priming could be related to the accumulation of key cellular proteins in their inactive state, which could be readily activated following exposure to biotic or abiotic stress speeding up the signal amplification cascade [33]. Another hypothesis on the molecular mechanism of priming suggested that epigenetic mechanisms get ready the defense genes in a permissive modified state facilitating quicker and more potent responses to subsequent attacks [34]. Additionally, several studies have shown the relevance of epigenetic mechanisms underlying priming phenomenon [17,18,35,36]. 

Rice (*Oryza sativa* L.) is an important food crop worldwide. In rice, priming was mainly used to improve seeds performance in terms of higher rate of germination and seedlings vigor under suboptimal environmental conditions. In the so-called “seed priming”, controlled hydration of seeds is used to break dormancy, speed germination and improve germination under stress conditions [37]. Recently, “seed priming” was also exploited to enhance the tolerance against various abiotic stresses including drought, submergence, salinity, chilling, and heavy metals in various plant species [38,39,40].

Recent developments in “omics” disciplines have opened up new perspectives to achieve a comprehensive understanding of biological processes related to stress responses in plants. In the post-genomic era, the enormous amounts of high throughput -omics data along with robust bioinformatics and data mining tools can potentially provide a global view on physiological processes triggered by stresses and also support the identification of novel signaling nodes in the plant defense signaling. Indeed, advances in transcriptomic, metabolomic and proteomic technologies allowed highlighting new hallmarks of biotic and abiotic stress responses in several plant species [41,42,43,44,45]. In particular, proteomics could be crucial to understanding physiological processes that are not accounted at genomic level. The few proteomic studies published so far on the priming role during environmental stresses identified key protein targets and signaling pathways, which are involved in mitigating negative effects of stress factors [19]. Recently, proteomics has been exploited to characterize the response of monocots to MeJA [46,47,48]. In particular, a proteomic analysis has suggested a role for MeJA in enhancing fungal disease resistance in rice [49]. Remarkably, to date there are not many studies highlighting the role of MeJA in protecting plants from wounding in rice. Mechanical wounding, which is induced by biotic (e.g., herbivore attack and pathogens infection) and abiotic (e.g., raining, wind, touching, and hailing) factors in plants, exists widely in nature [50]. Wounding stress is pretty deleterious since it can open the way to the invasion by microbial pathogens, providing nutrients to pathogens and facilitating their entry into the tissue and subsequent infection [51].

In this work, we investigated the impact of MeJA-induced priming on the efficacy of the rice plant response to mechanical wounding. The proteome profiling of MeJA-primed plants has been in-depth analyzed by using a combination of high throughput profiling techniques and bioinformatics tools. Moreover, Gene Ontology (GO) analysis has been carried out to obtain more comprehensive insight into the biological processes affected by MeJA, wounding and MeJA + wounding treatments. Moreover, we showed that low doses of MeJA prime plants for augmented level of a subset of proteins, upon wounding. Interestingly, while some of them are defense-related, others are involved in oxidative stress responses and photosynthesis. Finally, phenotypic analysis performed on primed and not primed rice plants strengthened the role of MeJA in protecting plants against potential oxidative stresses and photosynthetic alterations due to mechanical stress. To the best of our knowledge, this is the first study performed by shot-gun proteomics-based approach to investigate the role of MeJA as priming agent against wounding in rice.

## 2. Results and Discussion

**Figure 1:**
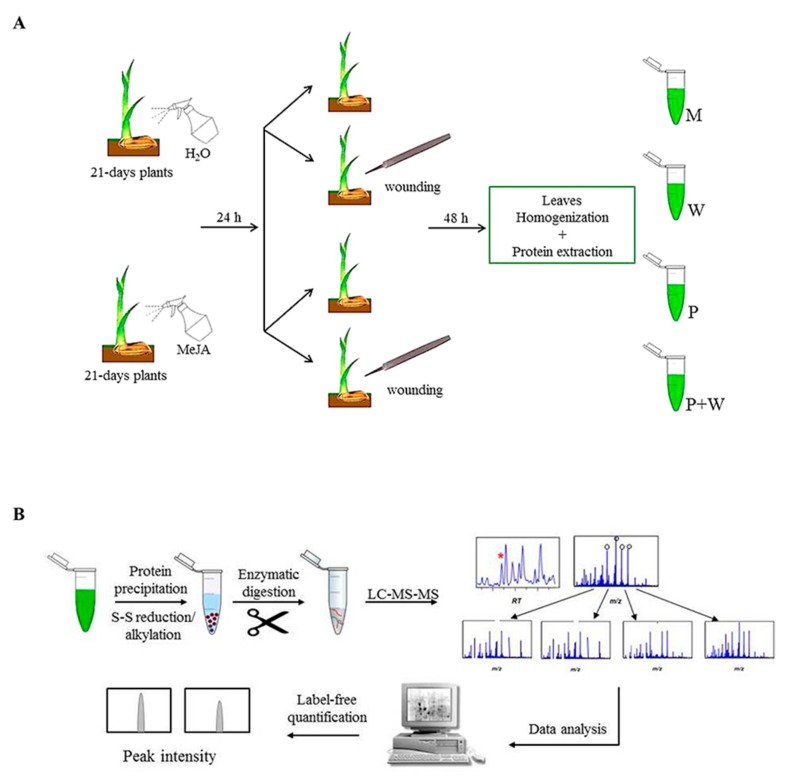
Experimental workflow. (**A**) Plant treatments. Mock (M), leaves after wounding (W), leaves primed with MeJA (P), and leaves primed with MeJA followed by wounding (P + W). (**B**) Protein treatment and proteomic analysis.

Jasmonates (JAs) are plant-specific signaling molecules that steer a broad set of physiological as well as defense processes. Pathogen attack and wounding caused by herbivores induce the biosynthesis of JAs, activating defense responses both locally and systemically [52]. To shed some light on the effect caused by MeJA-induced priming on defenses against herbivorous, we investigated the whole proteome changes of rice plants subjected to mechanical wounding following or not priming treatment by comparative proteomic analysis. An overview of the experimental workflow is shown in Figure 1.

To induce priming state, 21-day-old rice plants (3–4 leaves stage) were sprayed with 10 µM MeJA solution, a dose not able to induce direct defense response, as previously demonstrated [53]. Twenty-four hours after spraying, both mock and primed plants were wounded. Since plant response to MeJA is quite fast, the time gap elapsed between hormone treatment and wounding is reasonably enough to allow the establishment of the priming effect [53,54]. Leaf samples were harvested at 48 hours-post-wounding (hpw) and proteins were extracted from mock (M), wounded (W), primed with MeJA (P) and wounded after priming treatment (P + W) rice plants (Figure 1A). Comparative quantitative proteomic analysis (Figure 1B) was performed analyzing the proteome of the W, P and P + W rice leaves with respect to the plants grown under physiological conditions (M), allowing the identification of differentially expressed proteins (DEPs) in the treated samples. In the M sample, a total of 1417 proteins was identified, while in P, W, and P + W samples, the proteins detected were 1448, 1430 and 1447, respectively. Three biological replicates were performed and the numbers reported above encompass only the proteins overlapping between the three replicates. The differences in their levels were evaluated by label-free quantification approach, chosen to avoid excessive manipulation of samples and artifacts, using the MaxQuant software (Table S1).

### 2.1. MeJA Treatment Modulates Broad Spectrum Biological Processes 

The list of Differentially Expressed Proteins (DEPs) after MeJA treatment (P samples) compared to the mock is reported in Table 1.

Actually, listed DEPs are only those identified by at least three unique peptides, showing a level greater than two-fold or lower than two-fold (log_2_ fold change >1 or <−1, respectively) compared to the mock, and with a *p*-value ≤ 0.05. The analysis disclosed 32 proteins that underwent significant quantitative variations in plants treated by MeJA. Among these, 21 proteins showed a log_2_ fold change value greater than 1, indicating over-expression, whereas 11 proteins were found to be under-expressed with log_2_ fold change value less than -1, as compared to the mock.

**Figure 2:**
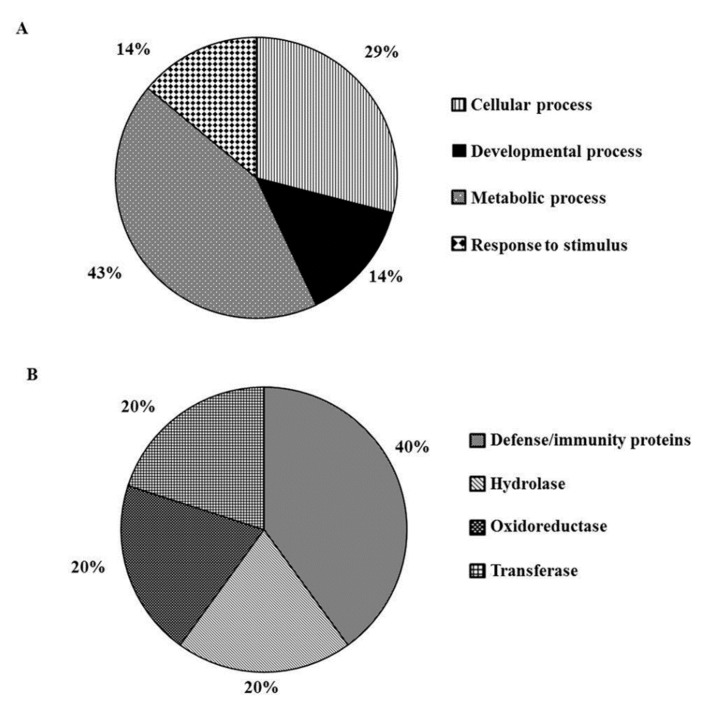
Functional classification of the 32 DEPs after MeJA treatment, using Protein Annotation Through Evolutionary Relationship (PANTHER) gene ontology (GO) analysis. The proteins were classified into (**A**) biological processes and (**B**) protein classes.

Gene Ontology analysis was performed by the Protein Annotation Through Evolutionary Relationship (PANTHER) software to classify DEPs into two major categories: biological processes and protein classes (Figure 2A,B, respectively).

GO analysis highlighted biological processes affected by MeJA treatment, largely represented by metabolic processes (43%) as well as cellular processes (29%) (Figure 2A). Moreover, our analysis revealed some proteins involved in developmental processes (14%). Indeed, it is well known from the literature that JAs are involved in the regulation of many developmental processes, including male fertility, fruit ripening, and root growth [52]. Within this group of proteins, we found a “probable cinnamyl alcohol dehydrogenase 8B” (OsCAD8B) (UniProt code Q6ERW9, Table 1), involved in lignin biosynthesis catalyzing the final step of the production of lignin monomers [55]. Interestingly, it has been reported that CAD genes are also stress-responsive [56]. The rice genome contains 12 different CAD genes distributed at nine different loci and expression patterns have been reported only for few of them; moreover, it has been hypothesized that the rice CAD genes could share similar expression profiles with orthologs in other plant species [55]. The OsCAD8B closest related gene is LpCAD1, characterized in *Lolium perenne*, which was found was found to be wound induced within six hours, but its level dropped down between 24–48 h [57]. According to this, in our experimental condition, OsCAD8B was found to be slightly down-regulated 48 hours after treatment with low doses of MeJA, but its induction at earlier time point after treatment cannot be ruled out. Our *in silico* functional characterization highlighted also that 14% of the input proteins belongs to the “Response to stimulus’’ group (Figure 2A). A representative protein of this category is germin-like protein 8-3 (UniProt code Q6YZZ7, Table 1) that resulted over-expressed in our experimental conditions and it is known to play a role in broad-spectrum disease resistance [58]. Members of the *Oryza sativa* 12 germin-like protein (OsGLP) gene cluster are located on chromosome 8 in the major-effect quantitative trait loci (QTL) for fungal blast resistance. In particular, proteins belonging to the OsGLP family were shown to contribute to disease resistance as silencing of several genes confers susceptibility to two distinct fungal pathogens, *Magnaporthe oryzae* and *Rhizoctonia solani*, the sheath blight pathogen [58]. In general, germins and germin-like proteins (GLPs) constitute a large plant gene family and they were first identified searching for germination-specific proteins in wheat (*Triticum aestivum*) [58,59]. They are present as glycoproteins often retained in the extracellular matrix by ionic bonds. Most are very stable oligomers [60,61]. They are structurally related to members of the cupin superfamily, that includes isomerases, sugar- or auxin-binding proteins, cyclases, dioxygenases, and monomeric or dimeric globulin seed storage proteins, such as phaseolin [62,63]. Germins and GLPs are known to play a wide variety of roles as enzymes, structural proteins, or receptors [60]. As Enzymes, germins have oxalate oxidase activity [64,65] and some GLPs have superoxide dismutase (SOD) activity [60], highlighting a role in defense responses since both of them can produce hydrogen peroxide (H_2_O_2_) in plants [66]. Some studies have demonstrated that germins and GLPs modulate plant responses to abiotic and biotic stresses [61,62]. Moreover, according to our findings, they are responsive to MeJA [67].

As shown in Figure 2B, DEPs are mostly “Defense/immunity proteins” (40%), whereas other protein classes are represented by Hydrolase, Oxidoreductase and Transferase (20% each). Among defense/immunity proteins are putative Pathogenesis-Related (PR) protein PRB1-2 (UniProt code Q8LMW8, Table 1) and Prb1 (UniProt code Q7XAD8, Table 1) belonging to the PR1 family, both over-expressed in our experimental conditions. PR1 family is a dominant protein group induced by pathogens and is commonly used as a marker for SA-related systemic acquired resistance (SAR) [68]. In rice it has been shown that Prb1 proteins were induced in roots of seedlings after salt stress or JA treatment, as well as in JA-treated stems or leaves [69,70,71,72]. Within the “Hydrolase” group a drought-induced gene, DIP3, encoding a chitinase III protein (UniProt code Q5WMX0, Table 1) was found over-expressed in our experimental condition. Chitinases (EC 3.2.1.14) catalyze the hydrolytic cleavage of the β-1,4-glycosidic bond in N-acetyl-glucosamine biopolymers largely found in chitin [73,74]. One of the physiological roles of plant chitinases is the protection against fungal pathogens by degrading chitin [75]. Remarkably, some chitinases do not show any antifungal activities [76]. Chitinases also respond to abiotic stress, and are involved in developmental processes or growth [74,77]. Notably, it has been demonstrated that treatment by JA induces the accumulation of chitinases in rice [78], according with our results. The protein class “Transferase” includes the protein o-methyltrasferase (UniProt code Q53LW0, Table 1), putatively involved in serotonin and melatonin biosynthesis, which was found strongly over-expressed in our experimental system. Melatonin (*N*-acetyl-5-methoxytryptamine) has been characterized as an important bioactive molecule that is not only an animal hormone, but also plays a role in plant growth and development [79]. Although significant advances in elucidating the physiological roles and biochemical pathways of melatonin in animals have been achieved, studies on melatonin in plants are at their infancy, but advancing rapidly [80]. Very recently, it has been reported that its functions in plants include also the ability to reduce susceptibility to diseases [81]. Our results corroborate the role of MeJA in rewiring a broad spectrum of biological processes in rice plants, even at low doses.

### 2.2. Wounding Induces Proteome Changes on Immunity-Related Proteins and Enzymes

The list of DEPs after wounding (W samples) compared to the mock is reported in Table 2.

**Figure 3:**
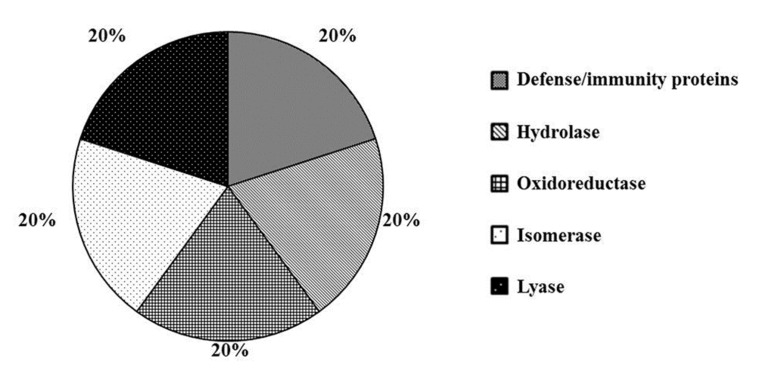
Functional classification of the 11 DEPs after wounding, using PANTHER gene ontology (GO) analysis. The proteins were classified into protein classes.

The analysis disclosed 11 proteins subjected to significant quantitative variations in wounded plants. Among these, 10 proteins showed a log_2_ fold change value greater than 1, indicating over-expression, whereas only 1 protein showed a log_2_ fold change <−1, as compared to the mock. Analysis was performed by PANTHER software and DEPs were classified into the category of protein classes. As shown in Figure 3, DEPs can be grouped in the following functional groups: “Defense/immunity proteins”, “Hydrolase”, “Isomerase”, “Lyase” and “Oxidoreductase” (20% each).

The only protein significantly repressed by wounding is categorized into “Oxidoreductase” and it is represented by a putative isoflavone reductase (UniProt code Q9FTN5, Table 2). Isoflavone reductases are enzymes involved in the biosynthesis of isoflavonoid phytoalexins in plants. They play essential roles in response to several biotic and abiotic stresses and are restricted to the plant kingdom. Isoflavonoid phytoalexins are small anti-microbial compounds produced by plants upon pathogen attack, exposure to elicitor molecules, or other biotic and abiotic stresses [82]. In rice, an isoflavone reductase-like, OsIRL, was found to be regulated by phytohormones either positively through JA or negatively through SA and ABA [82]. Moreover, when produced in combination with JA, upon wounding or herbivory, ABA acts synergistically on the expression of the MYC branch of the JA response pathway, while it antagonizes the ERF branch, induced by JA and ET [6,83]. A role of ABA in defense against insects has been suggested also in Arabidopsis [84]. Moreover, ABA has been demonstrated to be involved in gene regulation in response to wounding; in fact, endogenous ABA levels rise in plants after mechanical damage, both locally and systemically [69]. In light of this and according to previous findings [82], we may speculate that in our experimental conditions the isoflavone reductase-like under study could be down-regulated after wounding due to the wound-induced increase of ABA.

The GO category “Defense/immunity proteins” includes the protein Prb1 (UniProt code Q7XAD8, Table 2). We previously found that Prb1 is over-expressed also by MeJA (Table 1) and we discussed about its role in plant defense. Accordingly, it has been demonstrated that PR1 gene family is over-expressed after wounding in rice [85]. Moreover, the Arabidopsis Prb1 ortholog (At2g14580) is also involved in response to wounding (source: TAIR). The Protein class “Hydrolase” includes DIP3, a chitinase III protein (UniProt code Q5WMX0, Table 2). In our hands we found DIP3 up-regulated also by MeJA treatment (Table 1) and this is not surprising since it is well known that chitinases are induced by different abiotic stresses such as salt, cold, osmosis and heavy metals. For instance, in Arabidopsis, chitinase activity is induced by heat shock, UV light, and wounding [74]. Our study suggests that proteome reprogramming induced by wounding involve a broad variety of proteins functionally related with immunity processes and mainly aiming to boost defense responses.

### 2.3. Combined MeJA Treatment and Wounding Affect the Level of Proteins Related to Defense Processes

The list of DEPs after MeJA treatment followed by wounding (P + W sample), as compared to the mock is reported in Table 3.

**Figure 4:**
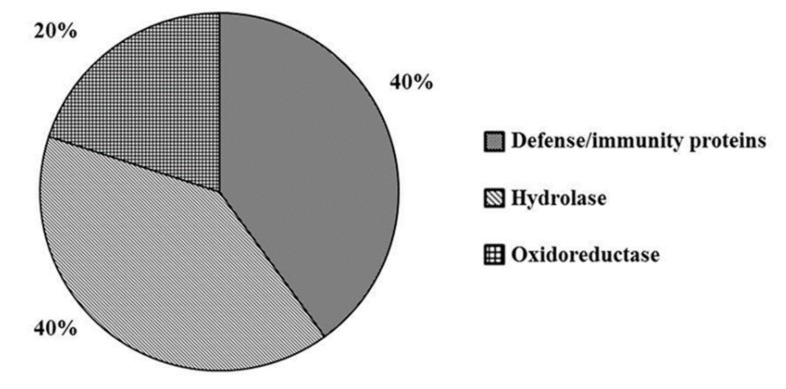
Functional classification of the 26 DEPs after MeJA + W, using PANTHER gene ontology (GO) analysis. The proteins were classified into protein classes.

The analysis disclosed 26 proteins that underwent significant quantitative variations with respect to mock. Among these, 24 proteins showed a log_2_ fold change value greater than 1, indicating over-expression, whereas 2 proteins were found to be under-expressed (log_2_ fold change value less than −1), as compared to the mock. Results of Gene Ontology analysis performed by PANTHER is shown in Figure 4. 

DEPs are classified into “Defense/immunity proteins” (40%), “Hydrolase” (20%) and “Oxidoreductase” (40%). The protein class “Defense/immunity proteins” includes the Pathogenesis-related proteins PRB1-2 (UniProt code Q8LMW8, Table 3) and Prb1 (UniProt code Q7XAD8, Table 3). As described above, both are significantly over-expressed by MeJA (Table 1), but only Prb1 was found significantly over-expressed also by wounding (Table 2).

Analogously, within the “Hydrolase” group, we disclosed DIP3 (UniProt code Q5WMX0, Table 3) also found over-expressed by MeJA (Table 1) and wounding (Table 2), implying that the double treatment is not essential for its induction. Within the “Hydrolase” group another representative protein is a purple acid phosphatase encoded by *NPP1* (UniProt code Q6ZI95, Table 3). Plant acid phosphatases are involved in phosphate acquisition and utilization and their synthesis is affected by developmental as well as environmental cues. Phosphate starvation induces *de novo* synthesis of extra- and intra-cellular acid phosphatases, that might be one of the strategies plants have evolved to cope with phosphate-limiting conditions [86]. Purple acid phosphatases have mainly been studied in Arabidopsis, especially for their response to phosphorus starvation. Induction at both mRNA or protein level in roots and in leaves under phosphate deficiency suggests that they may function in scavenging phosphate from the soil as well as recycling it within the plant [87]. Interestingly, purple acid phosphatases share similar transcriptional regulation features to *Arabidopsis Vegetative Storage Protein2* (*AtVSP2*) gene. Basically, *AtVSP2* is a gene induced by wounding, MeJA and insect feeding. Moreover, the defense function of AtVSP2 is correlated with its acid phosphatase activity [88]. In addition, the Arabidopsis ortholog of the purple acid phosphatase encoded by *NPP1*, *AtPAP27* (At5g50400), shared 65% of amino acid sequence identity with rice NPP1. By querying Genevestigator V3 [89] we found a strong induction of *AtPAP27* following MeJA treatment or wounding in different plant developmental stages, suggesting a similar role of rice NPP1 in plant defense mediated by MeJA and wound signaling.

Within the “Oxidoreductase” group, Os07g0664300 protein was found as differentially expressed (UniProt code Q0D3V1, Table 3). This protein belongs to the Short-chain Dehydrogenases/Reductases (SDR) family. SDR comprises a broad family of NAD(P)H-dependent oxidoreductases represented in plant kingdom. Functions of SDRs include many aspects of primary (chlorophyll biosynthesis, lipid synthesis, or degradation) and secondary (steroids, terpenoids, phenolics and alkaloids) metabolism. In analogy with animal SDRs, it may be rational to assume that several SDRs play a major role regarding hormone metabolism, including ABA biosynthesis [90]. Our results corroborate the evidence that MeJA and wounding signaling could overlap in inducing proteins with key roles in defense responses.

### 2.4. Priming-Regulated Proteins Correlate with Defense Processes

Priming mechanisms include the accumulation of proteins in an inactive form that are rapidly modulated upon exposure to stress, resulting in a more efficient and robust defense mechanism [34]. Our ultimate goal was to highlight proteins specifically affected by the priming treatment, i.e., all proteins that after MeJA treatment and subsequent wounding (P + W) showed a alevel greater than two fold (log_2_ fold change >1) compared to both wounding (W) and MeJA (P) single treatment and having a *p*-value ≤ 0.05. This comparison was performed in order to exclude the contribution of single treatments to the protein level occurred in the double treatment and to characterize molecules regulating plant priming as well as the potential interplay between them at proteome level. These proteins are listed in Table 4.

The protein carbonic anhydrase (UniProt code Q7F2G3) encoded by *Os01g0639900* gene, belongs to the large family of Carbonic Anhydrases (CAs). CAs are zinc metalloenzymes that catalyze the interconversion of CO_2_ and bicarbonate. CAs are ubiquitous in nature and they play essential roles in all photosynthetic organisms [91]. In plants, CAs are involved in various physiological processes such as photosynthesis, stomatal movement, development, amino acid biosynthesis, metabolism of nitrogen-fixing root nodules and lipid biosynthesis [91]. CAs are also involved in biotic and abiotic stress responses in both monocots and dicots [91]. In particular, many of them have been reported as involved in response against various pathogens and pests [92,93,94]. Moreover, there are evidences of CAs involvement in plant response to MeJA. Recombinant inbred lines of Arabidopsis resistant to the herbivore insect *Plutella xylostella* showed a limited oxidative stress, due to a 2-fold increase in abundance of AtbCA1 and AtbCA4 proteins [94]. Moreover, a proteomic study demonstrated that CA1 and CA2 from Arabidopsis are strongly up-regulated by MeJA [41].

The importance in restraining oxidative stress induced by (a)biotic cues is emphasized by the presence of ROS scavengers among the priming-regulated proteins disclosed in this study. Biotic and abiotic stresses can induce an oxidative burst, which is followed by rapid changes in hydrogen peroxide (H_2_O_2_) levels, leading to a variety of physiological responses in plants. Catalases (CATs) and peroxidases (Prxs) are heme enzymes that are able to detoxify H_2_O_2_, protecting cells from its toxic effects. In our study, a catalase encoded by *Os03g03910* (UniPROT code Q10S82) and a peroxidase encoded by *Os04g0688300* (UniProt code Q7XSU8) were found involved in priming phenomenon. Our peroxidase belongs to class III peroxidases which are glycoproteins located in vacuoles and cell walls [95]. They are part of a large multigenic family with 138 members in rice and 73 members in Arabidopsis [96]. Prxs belong to the PR9 family [97] and are involved in a broad spectrum of physiological processes, probably due to the high number of enzymatic isoforms (isoenzymes) and to the versatility of their enzyme-catalyzed reactions [95]. Indeed, plant Prxs are involved in lignin and suberin formation, cross-linking of cell wall components, auxin metabolism, phytoalexin synthesis and metabolism of ROS [95]. Prxs ability to catalyze the synthesis of bioactive plant products enables them to exert a role in plant defense. For example, Prxs are induced in host plant tissues by pathogen infection and are expressed to limit cellular spreading of the infection through the formation of structural barriers [95]. The stress-induced expression of Prx is conferred by the nature of the 5’ flanking regions of the genes that contain many different potential stress-responsive cis-elements [96]. According with our results, it has been widely reported that JA, MeJA and beneficial microbes with priming effects positively regulate *prx* gene expression [23,53,98].

Catalases (CATs) are major antioxidant enzymes primarily located in peroxisomes that detoxify hydrogen peroxide, produced from various metabolic reactions and environmental stresses, into oxygen and water [99]. Studies indicates that catalases play an important role in plant defense, aging, and senescence [100]. Furthermore, CATs are involved in the resistance of plant cell wall and they also act as a signal for the induction of defense genes playing a crucial role in maintaining active the defensive responses [101]. In rice, three classes of CATs have been identified as CatA, CatB, CatC, which are involved in environmental stress response, root growth, and photorespiration, respectively [102]. Interestingly, CATs are also involved in resistance against insects. It has been demonstrated that aphid resistance in tobacco plants infested with *Bemisia tabaci* nymphs is associated with enhanced antioxidant activities in which CAT may play a dominant role. Moreover, a proteomic study highlighted that CAT2 and CAT3 from Arabidopsis were strongly up-regulated by MeJA [41]. It has been shown that MeJA mediates intra- and inter-plant communications and modulates plant defense responses, including antioxidant systems [103]. In our systems, up-regulation of proteins involved in ROS scavenging corroborate the evidence that priming-induced plant resistance can be triggered by activation of redox-sensitive genes, as previously found [103]. 

Among the protein significantly involved in the priming phenomenon there is also a “Putative ATP synthase gamma chain 1, chloroplast (H(+)-transporting two-sector ATPase/F(1)-ATPase/ATPC1)” (UniProt code Q84NW1) encoded by *Os07g0513000* gene, belonging to the ATP synthesis-coupled proton transport. ATP synthase is a greatly conserved enzyme catalyzing the synthesis of ATP from ADP and phosphate through a flux of protons over an electrochemical gradient. Interestingly, proteolytic fragments of chloroplastic ATP synthase have been found to mediate plant perception of herbivory through the induction of volatile, phenylpropanoids, protease inhibitors and hormones, including MeJA [104].

**Figure 5:**
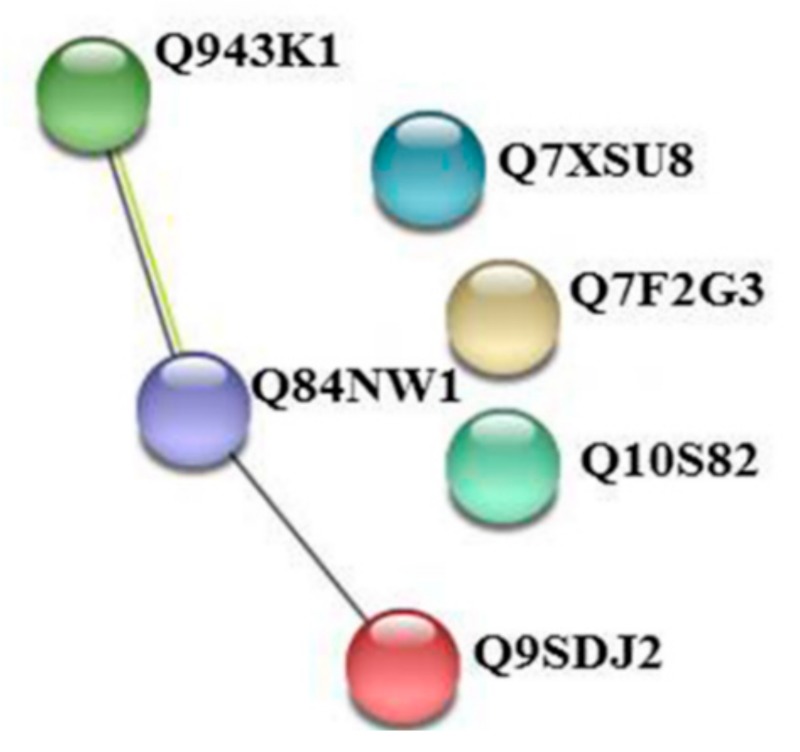
Interaction map of priming-regulated proteins. Network was built by using STRING 10.5 software, at 0.4 confidence level. Prediction was performed on proteins listed in Table 4.

By using the freely available STRING program, we unraveled the interaction pattern of proteins involved in the priming phenomenon (Figure 5).

Analyzing the STRING output, we found out that ATP synthase (UniPROT code: Q84NW1) interacts with Magnesium-protoporphyrin IX monomethyl ester [oxidative] cyclase (UniPROT code: Q9SDJ2), encoded by *ZIP1* gene and with a Putative photosystem II subunit PsbS (UniPROT code: Q943K1), encoded by *Os01g0869800* gene. Mg-protoporphyrin IX monomethyl ester cyclase is involved in the chlorophyll biosynthetic pathway [105]. The PsbS protein is a key component in the regulation of non-photochemical quenching (NPQ) in the photosynthesis of higher plant. The PsbS subunit of photosystem II (PSII) plays a crucial role in pH- and xanthophyll-dependent nonphotochemical quenching of excess absorbed light energy, thus contributing to the defense mechanism against photo-oxidative damage [106]. Taken together, our results highlighted that the MeJA priming brings about up-regulation of proteins involved in ROS scavenging and photosynthesis. This suggests an inter-pathway crosstalk between ROS, phytohormone signaling and photosynthesis that allows plants to efficiently respond to stress inputs, as previously reported [107]. It is worthwhile mentioning that the increased photosynthesis rates suggest a boost of primary metabolism probably due to the need of energy and carbon skeletons necessary for the synthesis of secondary metabolites. In general, it has been suggested that alterations in primary metabolism allow the plant to tolerate herbivores while minimizing impact on fitness traits. Therefore, alterations in the levels of key primary metabolites might themselves have the potential for a defensive mode of action.

### 2.5. MeJA Protects Plants from Effects of Wounding-Triggered H_2_O_2_ Production

**Figure 6:**
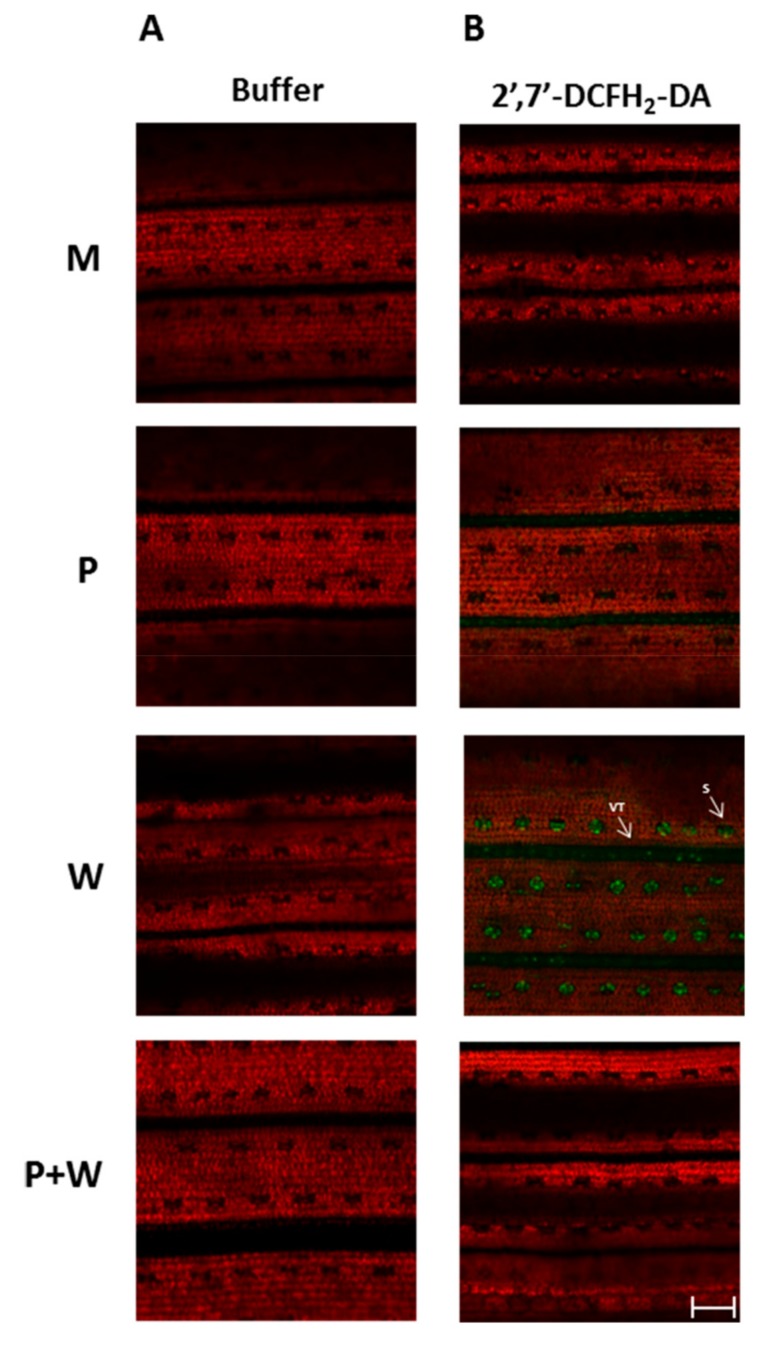
Detection of H_2_O_2_ in rice leaves using buffer (negative technical control) (**A**) or 2′,7′ DCFH_2_-DA (**B**). Detection was performed in rice leaves sprayed with a mock solution (M), with 10 μM MeJA solution (P), wounded (W), or sprayed with 10 μM MeJA and wounded (P + W) and harvested at 48 h-post-wounding. Fluorescence was observed under an LSM 710 confocal microscope with Planneofluoar 40/1.30 objective. Two laser excitations lines were used (i.e., 488 for probe detection and 563 nm for chlorophyll autofluorescence). S: stomata. VT: vascular tissue. Bar corresponds to 200 μm.

It is widely recognized that biotic and abiotic stresses induce ROS production in plant cells. Under adverse conditions ROS may play two very different roles: activation of signaling leading to defense responses or exacerbating damage. In fact, ROS can have a deleterious effect on cells since they can modify biomolecules such as nucleic acids, proteins, and lipids leading to cell damage and death [108]. At basal level, hydrogen peroxide (H_2_O_2_), the simplest peroxide recognized as ROS, plays important roles in several developmental and physiological processes When H_2_O_2_ accumulates in response to biotic and abiotic stresses, it is responsible of several phenomena, including stomatal closure and cell death [109]. Our aim was to highlight the presence of H_2_O_2_ in wounded rice leaves and to verify if MeJA can affect wounding-dependent H_2_O_2_ production exerting a priming role. To this end, three-week-old rice leaves from M, P, W and P + W plants were incubated with 2′,7′-dichlorofluorescein diacetate (2′,7′-DCFH_2_-DA) to detect the presence of H_2_O_2_. This compound exerts its function into the cytoplasm where is deacetylated by intracellular esterase and subsequently oxidized by H_2_O_2_ producing the green fluorescent dye dichlorofluorescein (2′,7′-DCF). A negative control is represented by leaves incubated with buffer only. Fluorescence was detected with a confocal microscope. The green fluorescence of the probe was revealed using a 488 filter whereas the auto-fluorescence of the chlorophyll was detected using a 563 nm filter. The experiment was performed three times independently and representative results are shown in Figure 6.

The negative control showed the red fluorescence due to the chlorophyll (Figure 6A). In panel B, wounded leaves exhibited very strong green fluorescence due to 2′,7′-DCF highlighting the presence of high levels of H_2_O_2_ localized at the level of stomata and vascular tissue. Noteworthy, in P + W green fluorescence was not observed anymore, suggesting a beneficial effect of the hormone in protecting plants from oxidative damage. It has been shown that H_2_O_2_ participates as a pivotal signal messenger in response to wounding in several species, including rice [110]. It is conceivable that ABA could represents one of the stimuli triggering H_2_O_2_ production during wounding [111]. The accumulation of H_2_O_2_ detected in stomata could depend on ABA-induced stomata closure, a phenomenon that exert a protection of leaves against further damage by subsequent threats [108,110]. Additionally, the presence of this compound in the vascular tissue could be due to its nature since it could migrate from the synthesis site to the neighboring vascular tissues or leaves, exerting a defense role against biotic agents [50]. Unfortunately, H_2_O_2_ levels are usually high under stress conditions so its effect as strong toxic oxidant agent could lead to cell damage or cell death since the ROS scavenging is compromised [112]. These latter considerations strengthen the role of MeJA in ameliorating plant cell life during adverse conditions.

### 2.6. MeJA Protects Plants from Photosynthetic Damage

**Figure 7:**
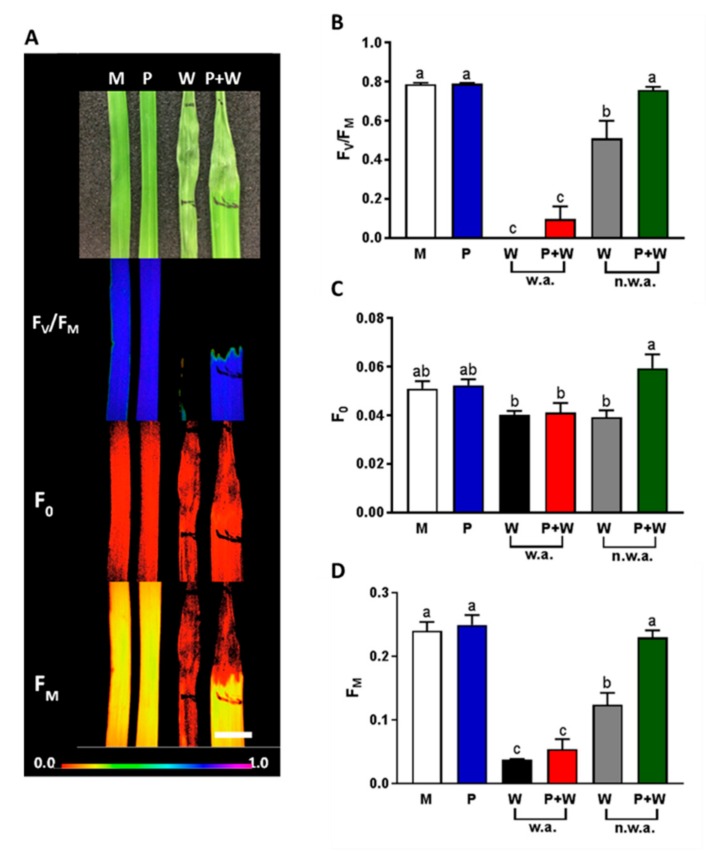
Measurement of chlorophyll fluorescence. Representative images of rice leaves mock (M); primed with MeJA (P); wounded (W); primed and wounded (P + W) in brightfield, maximum quantum efficiency of Photosystem II (F_V_/F_M_), minimal (F_0_) and maximal fluorescence (F_M_) (panel **A**). The color bar below shows the range of fluorescence values. Scale bar: 5 mm. Means (*n* = 5) ± SE of F_V_/F_M_ (**B**), F0 (**C**) and FM (**D**) values are shown. In all bar panels, white bars represent M, blue bars P, black bars W (w.a. = wounded area)), red bars P + W (w.a.), grey bars W (n.w.a = near wounded area), green bars P + W (n.w.a.). A one-way ANOVA followed by Tukey’s test was performed to define statistical significance (*p*  <  0.05) of differences among means. Data not sharing the same letters are statistically significantly different.

Chlorophyll fluorescence is a non-destructive method used to study plant photosynthetic performance in response to biotic and abiotic stresses [113] In this study, we used the chlorophyll fluorescence imaging tool in order to evaluate the effect of MeJA in wounded and not wounded leaves (Figure 7).

To this purpose, three-week-old rice leaves from M, P, W and P + W plants were imaged 48 hours after wounding. We measured the maximum quantum yield of photosynthesis, F_V_/F_M_, as a plant stress indicator whose decline refers to a compromised photosynthetic performance [114]. The maximum quantum yield of photosynthesis in M and P leaves was around a value of 0.8, indicating healthy leaves (Figure 7B). P + W leaves, compared to W, showed a reduced damaged area (Figure 7A) and a higher maximum quantum yield of photosynthesis (Figure 7B). In Figure 7A, the healthy part of the leaves fluoresced whilst the damaged part appeared dark. In the wounded area (w.a.) F_V_/F_M_ dropped to zero due to very low F_M_ values comparable to F_0_ (Figure 7B–D). In the regions near the wounded area (n.w.a.), P + W showed a F_V_/F_M_ similar to M and P whereas in W it was significantly decreased (Figure 7B). 

Other studies already showed a reduced chlorophyll fluorescence in wounded leaves [115] after herbivorous insect attack [116] and fungal infection [117,118]. The decrease in F_V_/F_M_, in our case, is due to low F_M_ values. F_M_ reduction can be related to photoinactivation of photosystem II (PSII) reaction centers or changes in PSII fraction due to modifications in thylakoid membrane structure and organization [119,120].

Our data suggests that MeJA treatment protects PSII reaction centers and maintain structural integrity of chloroplast, as already reported in salt stress conditions [121,122]. Therefore, we can put forward the hypothesis that low doses of MeJA configure the priming condition and that the hormone can exert this action inducing proteins that are able to reduce the damaged area and to protect the photosynthetic system.

## 3. Materials and Methods

### 3.1. Plant Material and Treatments

Rice seeds (*Oryza sativa* spp. *Japonica* cv. Carnaroli), supplied by Ente Nazionale Risi (Milano, Italy), were surface sterilized using 10% (*v*/*v*) H_2_O_2_ solution for 10 min. Seeds were washed with 70% (*v*/*v*) EtOH solution for 5 min, and soaked in water overnight. After incubation at 37 °C for 2 days on sterile water-imbibed filter paper, coleoptiles were transferred into alveolar trays and grown in hydroponic culture in Yoshida nutrient solution, in a growth chamber under the following conditions: 28°C, 14 h light / 23°C, 10 h dark, with 60% ± 5% relative humidity.

Three-week-old rice plants (3–4 leaves stage) were sprayed with 10 μM MeJA (Sigma; St. Louis, MO, USA) and 1% (*v*/*v*) TWEEN 20 solution to induce priming. Mock plants were sprayed with sterile water and 1% (*v*/*v*) Tween 20 solution only. Each plant was sprayed, making sure that droplets were uniformly distributed. Both mock and primed plants were wounded 24 h after MeJA treatment squeezing the leaves at the base and in the middle with a clamp and scraping the epidermal layer with carborundum in three different areas equally spaced over the length of the leaf. Leaf samples were harvested 48 hours-post-wounding (hpw) and homogenized by grinding with a pestle under continuous addition of liquid nitrogen.

### 3.2. Protein Sample Preparation

Grinded leaves were suspended in a lysis buffer containing 10% TCA in acetone and 10 mM DTT, left for 2 h at −20°C and then centrifuged at 13500 rpm for 14 min at 4°C. Pellet was washed in acetone, containing 10 mM DTT, 2 mM EDTA and 1 mM PMSF and centrifuged again under the same conditions. The obtained pellet was dried in Speed Vac Concentrator (Savant, ThermoFisher Scientific, Waltham, MA, USA).

Samples were solubilized in 100 mM Tris-HCl, pH 8.5, containing 8 M urea and 7.5 mM DTT, and sonicated by using 2 min-cycles (6 times) at 40 KHz and 4°C. Samples were subsequently centrifuged at 45,000 rpm for 10 min at 4°C. Protein quantification was conducted by BCA assay (Thermo Scientific, Rockford, IL, USA) in triplicate. Disulfide bridges reduction was performed by 10 mM DTT for 45 min at 30°C. Alkylation was obtained by 50 mM 2-iodoacetamide for 20 min, under dark. Protein digestion was performed by treating the diluted samples with two proteases. LysC digestion (LysC Mass spectrometry grade, WAKO, Neuss, Germany) was carried out by using an enzyme to protein ratio of 1:100, with an incubation of 4 h at 37 °C. The resulting digestion mixture was treated with Trypsin (Promega, Fitchburg, WI, USA) by using an enzyme to protein ratio of 1:50, incubating the samples overnight at 37 °C. Reactions were stopped by adding TFA to a final concentration of 0.5% and the mixture was desalted by RP-HPLC with a Zorbax column C18 eluted with a methanol gradient from 2 to 40% in 8 min, at a flow rate of 0.6 mL/min. Eluates were dried in Speed Vac Concentrator (Savant).

### 3.3. Proteomic Profiling and Data Analysis

LC-MS/MS analyses were conducted with a LTQ-Orbitrap XL mass spectrometer (ThermoFisher Scientific, Waltham, MA, USA) coupled online with a nano-HPLC Ultimate 3000 (Dionex−ThermoFisher Scientific, Waltham, MA, USA) using a 10 cm pico-frit capillary column (75 µm Internal diameter (I.D.), 15 µm tip, New Objective) packed in-house with C18 material (Aeris Peptide 3.6 um XB-C18, Phenomenex, Torrance, CA, USA). Peptides were eluted with a linear gradient from 3 to 50% of acetonitrile containing 0.1% formic acid in 160 min at a flow rate of 250 nL/min. The capillary voltage was set at 1.2 kV and the source temperature at 200°C. For the analysis a full scan at 60,000 resolution on the Orbitrap was followed by MS-MS fragmentation scans on the four most intense ions acquired with collision-induced dissociation (CID) fragmentation in the linear trap (data-dependent acquisition - DDA). For each analysis, about 1 µg of protein extract was used. Protein identification and quantification was performed by the software MaxQuant [123]. For each analysis, three biological replicates were analyzed. The database used for protein identification was the *Oryza sativa* section of the Uniprot database (version 20150805). Enzyme specificity was set to trypsin with 2 missed cleavages. The mass tolerance window was fixed to 20 ppm for parent mass and to 0.5 Da for fragment ions. Carbamidomethylation of cysteine residues was set as fixed modification and methionine oxidation as variable modification. Proteins were filtered with a false discovery rate (FDR) ≤ 0.01. Data from different samples were compared using a T-test with a level of significance of 95% (*p* ≤ 0.05). For Gene Ontology analysis, data were analyzed by the PANTHER version 11.0 [124]. The PANTHER classification system combines gene function, ontology, pathways and statistical analysis tools allowing to analyze large-scale data from sequencing, proteomics or gene expression data. PANTHER is based on 82 complete genomes data organized in gene families and subfamilies. Genes are classified according to their function, with families and subfamilies annotated with ontology terms (Gene Ontology (GO) and PANTHER protein classes). STRING analysis (Search Tool for the Retrieval of Interacting Genes/Proteins, http://string.embl.de/) has been carried out using STRING-10.5 server to predict the protein-protein interaction of priming targets [125]. STRING database employs a mixture of prediction approaches and a combination of experimental data (neighborhood, gene fusion, co-expression, experiments, databases, text mining, co-occurrence). Network was completed at 0.4 confidence level.

### 3.4. ROS Detection in Rice Leaves 

ROS detection was performed as previously described [126]. Briefly, H_2_O_2_ production was revealed by the specific probe 2′,7′-dichlorofluorescein diacetate (DCFH_2_-DA; Sigma Aldrich, St. Louis, MO, USA), which is rapidly oxidized to highly fluorescent dichlorofluorescein (DCF) in the presence of H_2_O_2_. Three-week-old rice plants were treated with MeJA or with sterile water (mock), as described above. Both mock and primed plants were wounded 24 h after MeJA treatment. For each treatment, two leaves from five plants were collected at 48 hpw Half number of leaves was incubated in a solution containing 20 mM DCFH_2_-DA in 10 mM Tris-HCl (pH 7.4) for 45 min under dark. The remaining half leaves was incubated in 10 mM Tris-HCl (pH 7.4) only, under the same conditions (negative technical control). After staining, leaves were washed three times in fresh buffer for 10 min and mounted on slides A LSM 710 confocal microscope (Carl Zeiss Microscopy GmbH, Jena, Germany) with Planneofluoar 40/1.30 objective, was used to detect the fluorescence. Two laser excitations lines were used (i.e., 488 nm for probe detection and 563 nm for chlorophyll auto-fluorescence). Data were managed using Image J software 1.46r (http://rsbweb.nih.gov/ij/) (LOCI, University of Wisconsin, Wisconsin, UW, USA). The experiment was performed three times independently.

### 3.5. Chlorophyll Fluorescence 

Plants were dark-adapted for 30 min before chlorophyll fluorescence measurements. The minimal fluorescence (F_0_), maximal fluorescence (F_M_) and maximum quantum efficiency of Photosystem II (F_V_/F_M_ = F_M_ − F_0_/F_M_) were measured in single leaves using an Imaging Pam M-series fluorimeter (Heinz Walz GmbH, Effeltrich, Germany) [116]. ImagingWin software (Heinz Walz GmbH, Effeltrich, Germany) allowed to select regions of interest in wounded leaves and to refer measurements to wounded (w.a.) and near wounded areas (n.w.a.). Data are shown as means ± standard errors (SEs). The normality of data distribution was tested using the Shapiro–Wilk Normality Test. Significant differences (*p* < 0.05) were analyzed using a one-way analysis of variance (ANOVA) followed by Tukey post-hoc test. SigmaPlot was used for the analysis (Systat Software Inc., San Jose, CA, USA).

## 4. Conclusions

Priming encompasses accumulation of latent signaling components that are quickly activated when plants are exposed to a stress. Therefore, it is interesting to exploit comparative proteomic analysis in plants treated with chemical priming agents before they encounter stress conditions. Our results strengthen the awareness that LC-MS/MS-based proteomic approach is an exceptional analytical tool for a better understanding of plant defense molecular mechanisms and of the proteome reprogramming modulated by different treatments. Using this approach, we highlighted proteins involved in ROS signaling and photosynthesis that could cooperate in regulating priming-dependent defense responses. Some of the currently identified proteins had previously been shown to play a role in defense responses; however, our study revealed a role of MeJA-priming in protecting rice plants from mechanical damages. In the future, it would be interesting to further investigate the exact role of these proteins in priming phenomenon.

## Figures and Tables

**Table 1:** Differentially expressed proteins (DEPs) after MeJA treatment (P), compared to mock (M).

UniProt Code	MSU ID Code	Protein Name	Log_2_ Fold-Change	*p*-Value
Q10D65	LOC_Os03g52860	Lipoxygenase Linoleate 9S-lipoxygenase 2	6.66	0.018
Q53LW0	LOC_Os11g20160	O-methyltransferase	4.59	0.013
Q01HV9	no code	Arginine decarboxylase	3.67	0.005
Q0JR25	LOC_Os01g03360	Bowman–Birk type bran trypsin inhibitor	3.25	0.014
B7E4J4	LOC_Os05g31750	Os05g0382600	2.98	0.02
Q5WMX0	LOC_Os05g15770	DIP3	2.58	0.002
Q5Z678	LOC_Os06g47620	IAA-amino acid hydrolase ILR1-like 6	2.41	0.037
Q8LMW8	LOC_Os10g11500	Os10g0191300 protein (putative PRB1-2)	2.26	0.022
Q5U1I3	no code	Peroxidase	2.24	0.01
Q8S3P3	LOC_Os04g56430	DUF26-like protein	2.23	0.018
Q69JF3	LOC_Os09g36700	Os9g0538000	2.14	0.016
Q7XAD8	no code	Os07g0126400 protein (putative Prb1)	2.07	0.024
Q5ZCA9	no code	Bowman–Birk type bran trypsin inhibitor (Fragment)	2.0	0.008
Q6YZZ7	LOC_Os08g08970	Germin-like protein 8-3	2.0	0.015
Q75T45	LOC_Os12g36830	Os12g0555000 (root PR10)	1.77	0.014
Q8L6H4	LOC_Os03g32314	Allene oxide cyclase, chloroplastic	1.72	0.05
Q6YXT5	LOC_Os08g02230	Os08g0114300 protein	1.58	0.02
Q33E23	LOC_Os04g45970	Glutamate dehydrogenase 2, mitochondrial	1.33	0.024
Q40707	LOC_Os12g36880	PBZ1	1.26	0.01
B9F4F6	no code	Citrate synthase	1.1	0.046
Q9ATR3	no code	Glucanase	1.07	0.0012
Q0JG75	LOC_Os01g71190	Photosystem II reaction center Psb28 protein	−1.09	0.002
C5MRM9	no code	PsbA (Fragment)	−1.09	0.019
A2YVX9	no code	Putative uncharacterized protein (Germin-like protein 8-14)	−1.09	0.044
Q5QLS1	LOC_Os01g47780	Arabinogalactan protein-like	−1.1	0.047
B7EKW3	LOC_Os07g26690	Aquaporin	−1.18	0.03
Q5Z5A8	LOC_Os06g51330	Photosystem II stability/assembly factor HCF136 chloroplastic	−1.32	0.045
Q6ERW9	LOC_Os09g23540	Probable cinnamyl alcohol dehydrogenase 8B	−1.36	0.041
Q7F8S5	LOC_Os02g09940	Peroxiredoxin-2E-2, chloroplastic	−1.64	0.029
B9FY06	LOC_Os07g38300	Ribosome-recycling factor, chloroplastic	−1.89	0.014
H2KW47	LOC_Os11g13890	Chlorophyll A-B binding protein, chloroplastic	−1.94	0.014
J3RG68	no code	Photosystem I iron-sulfur center	−2.06	0.013

**Table 2:** Differentially expressed proteins (DEPs) after wounding, compared to mock.

UniProt Code	MSU ID Code	Protein Name	Log_2_ Fold-Change	*p*-Value
Q306J3	LOC_Os12g14440	Dirigent protein	5.11	0.001
Q10D65	LOC_Os03g52860	Linoleate 9S-lipoxygenase 2	4.17	0.019
Q75T45	LOC_Os12g36830	Os12g0555000 (root PR10)	3.52	0.001
Q945E9	LOC_Os03g18850	JIOsPR10	2.38	0.011
Q40707	LOC_Os12g36880	PBZ1	2.04	0.035
Q5WMX0	LOC_Os05g15770	DIP3	1.63	0.001
Q8S3P3	LOC_Os04g56430	DUF26-like protein	1.63	0.012
Q5ZCA9	no code	Bowman–Birk type bran trypsin inhibitor (Fragment)	1.32	0.007
Q0JR25	LOC_Os01g03360	Bowman–Birk type bran trypsin inhibitor	1.2	0.025
Q7XAD8	no code	Os07g0126400 protein (putative Prb1)	1.14	0.036
Q9FTN5	LOC_Os01g01660	Os01g0106400 (putative isoflavone)	−1.56	0.044

**Table 3:** Differentially expressed proteins (DEPs) after MeJA treatment followed by wounding, compared to mock.

UniProt Code.	MSU ID Code	Protein Name	Log_2_Fold-Change	*p*-Value
Q10D65	LOC_Os03g52860	Linoleate 9S-lipoxygenase 2	5.3	0.015
Q5U1I3	no code	Peroxidase	5.1	0.039
Q306J3	LOC_Os12g14440	Dirigent protein	4.3	0.005
Q8S3P3	LOC_Os04g56430	DUF26-like protein	3.2	0.01
Q01HV9	no code	Arginine decarboxylase	3.1	0.033
Q5WMX0	LOC_Os05g15770	DIP3	3.0	0.01
Q9ATR3	no code	Glucanase	2.9	0.043
B7E4J4	LOC_Os05g31750	Os05g0382600	2.7	0.016
Q10N98	LOC_Os03g16950	33 kDa secretory protein, putative expressed	2.7	0.042
Q75T45	LOC_Os12g36830	Os12g0555000 (root PR10)	2.7	0.018
Q69JX7	LOC_Os09g36680	Drought-induced S-like ribonuclease	2.7	0.017
Q7XAD8	no code	Os07g0126400 protein (putative Prb1)	2.4	0.029
Q8LMW8	LOC_Os10g11500	Os10g0191300 protein (putative PRB1-2)	2.2	0.001
Q0JR25	LOC_Os01g03360	Bowman–Birk type bran trypsin inhibitor	2.2	0.037
Q69JF3	LOC_Os09g36700	Os09g0538000	2.1	0.008
Q6YXT5	LOC_Os08g02230	Os08g0114300 protein	1.9	0.008
Q5ZCA9	no code	Bowman–Birk type bran trypsin inhibitor (Fragment)	1.8	0.005
Q8L6H4	LOC_Os03g32314	Allene oxide cyclase, chloplastic	1.7	0.031
Q5Z7J2	LOC_Os06g35520	Peroxidase	1.7	0.033
Q40707	no code	PBZ1	1.7	0.037
Q33E23	LOC_Os04g45970	Glutamate dehydrogenase 2, mitochondrial	1.6	0.013
Q6ZI95	LOC_Os08g41880	Purple acid phosphatase	1.6	0.036
Q0D3V1	no code	Os07g0664300 protein	1.6	0.046
B9F4F6	no code	Citrate synthase	1.6	0.024
S4U072	LOC_Os04g39150	OSJNBb0048E02.12 protein	−1.0	0.023
Q10A54	LOC_Os10g05069	Alpha-mannosidase	−2.3	0.047

**Table 4:** Priming-regulated proteins. Log2 fold-change after MeJA+wounding, compared to wounding (P + W/W), log_2_ fold-change after MeJA+wounding, compared to MeJA (P + W/P) and corresponding p-values are shown.

UniProt Code	MSU ID Code	Protein Name	Log_2_ Fold-Change (P + W/W)	*p*-Value	Log_2_ Fold-Change (P + W/P)	*p*-Value
Q7F2G3	LOC_Os01g45274	Carbonic anhydrase, chloroplast precursor, putative, expressed	1.48	0.006	1.33	0.002
Q943K1	LOC_Os01g64960	Chlorophyll A-B binding protein, putative, expressed	1.22	9.29 × 10^−5^	1.21	0.001
Q84NW1	LOC_Os07g32880	ATP synthase gamma chain, putative, expressed	1.13	0.015	1.32	0.027
Q9SDJ2	LOC_Os01g17170	Magnesium-protoporphyrin IX monomethyl ester cyclase, chloroplast precursor, putative, expressed	1.54	0.003	1.42	0.005
Q10S82	LOC_Os03g03910	Catalase domain containing protein	1.33	0.005	1.48	0.007
Q7XSU8	LOC_Os04g59190	Peroxidase precursor, putative, expressed	1.21	0.049	1.14	0.028

## Supplementary Materials

Supplementary materials can be found at https://www.mdpi.com/1422-0067/20/10/2525/s1. Table S1. List of proteins identified by MaxQuant analysis, using the database Uniprot, taxonomy *Oryza sativa* (version 20150805). Enzyme specificity was set to trypsin with 2 missed cleavages. The mass tolerance window was set to 20 ppm for parent mass and to 0.5 Da for fragment ions. Carbamidomethylation of cysteine residues was set as fixed modification and methionine oxidation as variable modification. Content of columns: A, identified proteins by Entry name; B, identified proteins and other relevant information for the identification; C-F, unique peptides media relative to the three replicated of samples M, W, P, P + W; G-I, label-free intensities media relative to three replicates, standard deviation and percentage of coefficient variation of sample M; J-L, label-free intensities media relative to three replicates, standard deviation and percentage of coefficient variation of sample W; M-O, label-free intensities media relative to three replicates, standard deviation and percentage of coefficient variation of sample P; P-R, label-free intensities media relative to three replicates, standard deviation and percentage of coefficient variation of sample P + W.

## Abbreviations

MeJA	Methyl-jasmonate
M	Mock
P	MeJA- treated plants
W	Wounded plants
P + W	Wounded plants after MeJA treatment
